# Interactions between Hair Cells Shape Spontaneous Otoacoustic Emissions in a Model of the Tokay Gecko's Cochlea

**DOI:** 10.1371/journal.pone.0011116

**Published:** 2010-06-15

**Authors:** Michael Gelfand, Oreste Piro, Marcelo O. Magnasco, A. J. Hudspeth

**Affiliations:** 1 Howard Hughes Medical Institute and Laboratory of Sensory Neuroscience, The Rockefeller University, New York, New York, United States of America; 2 Departament de Física and Institute for Cross-Disciplinary Physics and Complex Systems (IFISC), Spanish National Research Council (CSIC) - University of the Balearic Islands (UIB), Universitat de les Illes Balears, Palma de Mallorca, Spain; 3 Laboratory of Mathematical Physics, The Rockefeller University, New York, New York, United States of America; Mount Sinai School of Medicine, United States of America

## Abstract

**Background:**

The hearing of tetrapods including humans is enhanced by an active process that amplifies the mechanical inputs associated with sound, sharpens frequency selectivity, and compresses the range of responsiveness. The most striking manifestation of the active process is spontaneous otoacoustic emission, the unprovoked emergence of sound from an ear. Hair cells, the sensory receptors of the inner ear, are known to provide the energy for such emissions; it is unclear, though, how ensembles of such cells collude to power observable emissions.

**Methodology and Principal Findings:**

We have measured and modeled spontaneous otoacoustic emissions from the ear of the tokay gecko, a convenient experimental subject that produces robust emissions. Using a van der Pol formulation to represent each cluster of hair cells within a tonotopic array, we have examined the factors that influence the cooperative interaction between oscillators.

**Conclusions and Significance:**

A model that includes viscous interactions between adjacent hair cells fails to produce emissions similar to those observed experimentally. In contrast, elastic coupling yields realistic results, especially if the oscillators near the ends of the array are weakened so as to minimize boundary effects. Introducing stochastic irregularity in the strength of oscillators stabilizes peaks in the spectrum of modeled emissions, further increasing the similarity to the responses of actual ears. Finally, and again in agreement with experimental findings, the inclusion of a pure-tone external stimulus repels the spectral peaks of spontaneous emissions. Our results suggest that elastic coupling between oscillators of slightly differing strength explains several properties of the spontaneous otoacoustic emissions in the gecko.

## Introduction

Ears are for hearing, yet the auditory organs of most tetrapods not only receive sound, but also *emit* it. Humans and other tetrapods usually produce spontaneous otoacoustic emissions at several frequencies scattered throughout the range of audition [1],[2]. These signals are idiosyncratic and, in the absence of aural damage, remain stable for months and even years. Emissions occur regularly in a low-noise laboratory environment, but occasional animals persist in radiating audible tones even under everyday circumstances [3].

Spontaneous otoacoustic emissions are one manifestation of an active process that improves the ear's performance in three ways [reviewed in [4]–[6]]. First, the cochlea amplifies its mechanical inputs, by that means increasing auditory sensitivity one-hundredfold. The active process next augments the cochlea's frequency discrimination with respect to that expected of a passive mechanical resonator, thus facilitating the identification of sound sources. Finally, the active process confers compressive nonlinearity on the ear's responsiveness: although hearing operates at sound-pressure levels from 0 dB to 120 dB, a millionfold range in stimulus amplitude, the cochlea responds with vibrations and electrical signals that encompass only two orders of magnitude.

The active process stems from the metabolically powered exertions of the ear's sensory receptors, the hair cells. Each of these cylindrical epithelial cells is surmounted by a mechanoreceptive hair bundle, a cluster of 20–300 minute, upright, actin-filled rods called stereocilia. The hair bundle is bilaterally symmetrical and exhibits a monotonic increase in stereociliary length along one axis. Acoustic stimuli conveyed through the complex hydromechanical linkages of the cochlea impinge upon hair bundles and deflect them. The resultant shearing motion between adjacent stereocilia is sensed by mechanoelectrical transduction channels, which open when the hair bundle is pushed toward its tall edge and shut for oppositely directed deflections [reviewed in [7], [8]]. Hair bundles can also move actively; in the ears of nonmammalian tetrapods, this motion has been demonstrated to account for the amplification, tuning, and compressive nonlinearity associated with the active process [reviewed in [5], [6], [ and 9]]. Active hair-bundle motility plays a role in the active process of the mammalian cochlea as well [10]–[12]. In that organ, however, electrically induced contractions of the hair-cell somata are thought to dominate the active process [reviewed in [13]–[15]].

An individual hair cell can make only a miniscule contribution to an ear's spontaneous otoacoustic emissions [16]. Each observed emission must therefore represent the synchronous activity of numerous cells. How, then, are these oscillators coupled to one another? What determines the spacing of successive emission peaks? How are spontaneous otoacoustic emissions influenced by concurrent stimulation with pure tones? We have explored these issues with an abstract model based on the basilar papilla of the tokay gecko, an animal that produces profuse, robust, and readily recorded spontaneous otoacoustic emissions [17], [18].

## Results

### Structure of the gecko's basilar papilla

**Figure 1:**
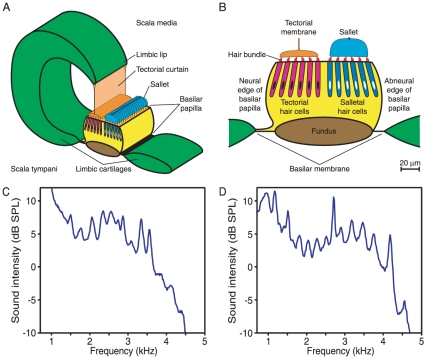
The gecko's cochlea and spontaneous otoacoustic emissions. A. A schematic view portrays one-tenth of the apical portion of the cochlea's sensory structure, the basilar papilla. The papilla rests upon the basilar membrane, a sheet of connective tissue suspended from the limbic cartilages and separating the scala media from the scala tympani. Acoustic stimulation creates a pressure difference between these liquid-filled compartments, thus setting the basilar papilla into up-and-down oscillation accompanied by side-to-side rocking. Above the papilla lie a continuous tectorial membrane and an array of about 170 sallets, 17 of which are depicted here, joined by a fine filament at their tops. B. A cross-section through the basilar papilla reveals one transverse row of hair cells. On the neural side of the papilla, the hair bundles of six tectorial hair cells insert into the continuous tectorial membrane, which hangs by the tectorial curtain from the limbic lip. On the opposite, abneural side of the papilla, the hair bundles of six salletal hair cells contact a sallet, a discrete tectorial structure about 20 µm in height. The remainder of the basilar papilla consists of supporting cells (yellow) atop a possibly cartilaginous fundus that provides a stiff beam extending the entire length of the papilla (A. Le Boeuf and A.J.H., unpublished observations). C. The power spectrum recorded from a tokay gecko portrays the typical features of the spontaneous otoacoustic emissions from this species, including a broad hump of power extending from less than 1 kHz to more than 4 kHz and a series of superimposed peaks. The frequency spacing between 12 pairs of adjacent peaks is 240±50 Hz. This particular spectrum displays several features of internal organization that were not observed routinely: a nearly constant interval of 281±6 Hz separates three pairs of peaks at both the low- and high-frequency ends of the spectrum, and in that the peaks at 3560 Hz and 4120 Hz are the second harmonics of those at 1780 Hz and 2060 Hz. D. A second spectrum shows the variability of emissions between geckos. In this instance, the interval between 13 pairs of peaks is 270±70 Hz. Local regions show even greater regularity: the spacings between the four peaks to the left of the larger central peaks are 229±8 Hz, whereas those to the right are separated by 277±3 Hz.

The acoustic receptor of the tokay gecko is the basilar papilla, a narrow strip of epithelial cells some 2000 µm long but only 100–130 µm in width [19], [20] (Figure 1A). The papilla rests upon the basilar membrane, a thin sheet of connective tissue supported around its perimeter by an elongated ring of cartilage, the limbus. In response to the pressure changes produced by sound stimulation, the basilar membrane oscillates up-and-down and carries the basilar papilla with it [21]–[23]. A band of nerve fibers entering the papilla along one edge defines the organ's neural side; the opposite side is termed abneural. The papilla encompasses about 2100 hair cells in some 240 irregular transverse rows [19]. The basal third of the organ contains hair cells responsive to sounds at frequencies below 1 kHz. The apical two-thirds, with which the present report deals, holds hair cells sensitive to higher frequencies; characteristic frequencies of auditory nerve fibers from this region range up to at least 5 kHz [24]. Hair cells within this apical region are arranged tonotopically, with frequency increasing continuously and exponentially toward the papilla's apex [25].

The apical portion of the basilar papilla displays two structural symmetries [19]. First, the array of hair cells is bisected longitudinally (Figure 1B). The hair cells in the neural half of the papilla are termed tectorial, for their hair bundles are surmounted by, and insert into, a continuous strip of extracellular gel called the tectorial membrane. The tectorial membrane is attached to a thin but uninterrupted curtain of tectorial material that hangs from the limbic lip, a wavelike ridge extending over the basilar papilla from one edge of the cartilaginous ring. On the papilla's abneural side, the hair bundles from each transverse row of up to seven hair cells insert into a discrete slab of extracellular material called a sallet. A thin strand interconnects the sallets longitudinally at their top edges. The second organizational symmetry is a longitudinal mirror plane within each complement of hair cells. In every transverse row across the basilar papilla, about half of the tectorial hair bundles are oriented with their vectors of excitability facing toward the papilla's neural edge, whereas the other half face the opposite, abneural edge. A similar symmetry pertains for the salletal hair cells.

The two classes of hair cells are likely to have distinct functions. The afferent nerve fibers that convey information into the brain contact the salletal hair cells [25], so those receptors clearly mediate the process of mechanoelectrical transduction. The tectorial hair cells, which lack any innervation, contribute to hearing presumably by participating in the active process through active hair-bundle motility [26]. Note that this assignment by no means precludes the possibility that salletal hair cells are also involved in the active process. Indeed, lizards of certain other species possess only salletal hair cells, yet display the spontaneous otoacoustic emissions diagnostic of an active process [27].

### Spontaneous otoacoustic emissions in the tokay gecko

The spontaneous otoacoustic emissions of the tokay gecko are striking in their ubiquity and structural regularity. When a sensitive microphone is used to record the sound pressure at the externally situated eardrum of a lightly anesthetized gecko, the power spectrum of the ensuing record shows the principal features of these emissions (Figure 1C, D). A broad hump of acoustic power characteristically extends from less than 1 kHz to almost 5 kHz, peaking near 3 kHz; background noise obscures any emissions at still lower frequencies. From this background protrude several distinct spectral peaks [17], [18], as many as fifteen depending on how peaks are defined. These emission peaks display varying degrees of regularity from animal to animal: some ears produce a comb of evenly spaced emissions of similar amplitude, whereas others show a ragged array of diverse peaks. In addition, the bandwidth of emissions at 3 dB below their peaks exceeds that in many other species, ranging from 44 Hz to 74 Hz [17]. By comparison, the comparable bandwidth for emissions recorded from humans is only 1 Hz [28]. The emission spectrum is sensitive to temperature [17] and to drugs that affect mechanoelectrical transduction [18]. The variability between individual lizards, and from recording to recording in a given animal, may additionally reflect differences in the placement of the microphone and in the level of anesthesia.

### Modeling hair-cell oscillators

A model intended to describe the gecko's spontaneous otoacoustic emissions must include descriptions both of the individual oscillators and of their interactions along the basilar papilla. Although detailed quantitative descriptions exist for hair-bundle oscillations in the bullfrog's sacculus [29]–[31], these equations cannot be applied directly to the gecko: the structures of the receptor organs differ dramatically between the two species, and emissions in the lizard occur at frequencies two orders of magnitude higher than the hair-bundle oscillations observed in the frog. In the absence of a detailed model appropriate for the gecko, and inasmuch as we wish to investigate the essential ingredients necessary to reproduce the observed emission spectra, we adopted a more qualitative strategy. Supposing first that the relevant oscillating unit consists not of a single hair cell but of a transverse row of hair cells operating at a single frequency [20], [32], and second that the qualities of spontaneous otoacoustic emissions depend primarily on interactions along the receptor organ, we chose a generic description of the individual oscillators.

The statistical properties of spontaneous otoacoustic emissions indicate that these signals originate from self-sustained oscillators in the ears both of lizards and of mammals [33], [34]. The van der Pol oscillator provides a simple and mathematically tractable representation of such an active element [35]. For example, this formulation reproduces the essential features of the interactions of spontaneous otoacoustic emissions with one another and with externally applied stimuli [36], [37]. A similar model simulates the changes in the synchronization of emissions to pure tones upon administration of aspirin [38]. A model based on a van der Pol oscillator emulates the synchronization of a spontaneous otoacoustic emission with a cubic distortion product [39]. Finally, such a model explains some features of the spontaneous otoacoustic emissions in another lizard, the Australian bobtail skink [40].

On the basis of measurements from other ears, the sallets of the tokay gecko are expected to move roughly ±50 nm during vigorous spontaneous oscillation [30]. For the stimulus frequencies represented in the apical portion of the basilar papilla, 1–71kHz, this motion corresponds to maximal velocities of 0.3–2.2 mm·s^−1^. Using the salletal width of about *w* = 50 µm as a characteristic dimension, these values suggest that the sallets operate at Reynolds numbers *Re* given by
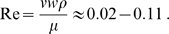
(1)


Here *ρ* is the density of the sallet and µ is the dynamical viscosity of the medium, both of which are assumed to equal the corresponding values for water. These values imply that the system is overdamped, a conclusion strengthened by the fact that each sallet experiences additional damping owing to its motion relative to that of the adjacent sallet on each side. Our formulation therefore excluded the effect of mass.

In our model, each of *N* units, denoted by the index *n*, was represented as a van der Pol oscillator characterized by the equations
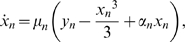
(2a)

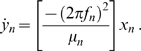
(2b)


Here *x* represents hair-bundle position; the variable *y* has no simple physical interpretation, but encompasses the adaptation process and other features of the hair bundle's internal dynamics [31]. This formulation incorporates the assumption, elaborated below, that the bundle's inertia can be neglected in the description of its dynamics. The elastic force that influences the hair bundle's position includes a cubic term that accords with the structure of the models for the bullfrog's hair bundle [9]. The parameters α, *f*, and µ determine respectively the amplitude, characteristic frequency, and nonlinearity of an individual unit's unforced oscillation.

We performed simulations of a one-dimensional array of oscillators each behaving according to these equations. The number of oscillators was ordinarily *N* = 110. The value of the parameter *α* was initially set to 1. The value of 2*πf_n_* varied exponentially along the chain from 1 to 2.97; the frequencies of successive oscillators thus differed by about 1%. Given the arbitrary timescale of the simulations, these frequencies are represented as falling between 1 kHz and 2.97 kHz to facilitate comparison with experimental data. Time intervals are presented in the corresponding units, generally in the millisecond range. The parameter µ also varied exponentially so that the ratio 2*πf*/µ remained constant at 5. Every unit thus differed from a harmonic oscillator by an equivalent amount; the unperturbed oscillations of the various units differed only in timescale. This choice of value for *µ* rendered the oscillations nearly sinusoidal. The parameter values employed in the specific simulations displayed below are listed in Table 1.

**Table 1:** Parameter values for simulations.

Figure	*N*	*α_n_*	*β*	*γ*	*γ*	*F*	Noise
**2A, 2B**	110	1	0.2	0	0	0	–
**3A, 3B**	110	1	0	1	0	0	–
**3C, 3D**	110	*n*/18 for *n* = 1–18	0	1	0	0	–
		1 for *n* = 19–92					
		(111-*n*)/18 for *n* = 93–110					
**3E**	110	*n*/18 for *n* = 1–18	0	0–2	0	0	–
		1 for *n* = 19–92					
		(111-*n*)/18 for *n* = 93–110					
**4A**	110	*n*(111-*n*)/3025	0	0.5	0	0	–
**4B**	210	1	0	1	0	0	–
**5A**	110	*n*/18 for *n* = 1–18	0	1	0	0	–
		1±0.03 for *n* = 19–92					
		(111-*n*)/18 for *n* = 93–110					
**5B**	110	*n*/18 for *n* = 1–18	0	1	0	0	+
		1 for *n* = 19–92					
		(111-*n*)/18 for *n* = 93–110					
**5C**	110	*n*/18 for *n* = 1–18	0	1	0	0	+
		1 for *n* = 19–92					
		(111-*n*)/18 for *n* = 93–110					
**6A, 6B**	110	*n*/18 for *n* = 1–18	0	1	0.2	1.8	–
		1 for *n* = 19–92					
		(111-*n*)/18 for *n* = 93–110					
**6C**	110	*n*/18 for *n* = 1–18	0	1	0–1	1.8	–
		1 for *n* = 19–92					
		(111-*n*)/18 for *n* = 93–110					
**6D**	110	*n*/18 for *n* = 1–18	0	1	0.2	0.6–3.6	–
		1 for *n* = 19–92					
		(111-*n*)/18 for *n* = 93–110					

In each simulation, the frequency 2*πf_n_* for the *n* ^th^ oscillator assumes the value 3^(*n*−1)/110^ and the ratio (2*πf_n_*)/*µ_n_* remains constant at 5. In the non-dimensionalized model, *N* and *β* have no units; the units of the remaining parameters are: *α_n_*, distance squared; *γ*, inverse time; *δ*, distance per time; *f_n_* and *F*, inverse time; and µ_n_, inverse distance squared times inverse time.

### The effect of viscous coupling

Previous research has demonstrated the importance of interactions between oscillators in the behavior of spontaneous otoacoustic emissions. A system comprising two asymmetrically coupled van der Pol oscillators recapitulates the responses of emissions to external suppressing tones [36], [37]. Suitable coupling between oscillators represents the response of the basilar membrane to sinusoidal stimulation [41]. Finally, the entrainment of weak oscillators by stronger ones can explain the minimal spacing between emission peaks in mammals and skinks [40], [42].

Although forces of many types might mediate coupling between adjacent oscillatory units, we focused on two that seem most likely to reflect physical elements present in the gecko's basilar papilla: viscous forces conveyed through short-range hydrodynamic interactions and elastic forces propagated along the tectorial structures of the papilla. Other mathematically possible interactions, such as inertial coupling, are difficult to explain in terms of a physical system and were not examined.

In the gecko's cochlea, viscous coupling between oscillators could arise from the mechanical activity of salletal hair cells. Because adjacent sallets are slabs of tectorial material separated by only a few micrometers, it is plausible that adjacent sallets interact hydrodynamically. Emissions are known to emerge from lizard species with only salletal hair cells [27], [43]–[45], so those cells are potentially capable of sustaining spontaneous oscillations. We therefore examined at the outset a model in which hair bundles are coupled by viscous drag.

We modeled the interactions between adjacent oscillators by modifying Equation 2a with a term proportional to the laplacian of velocity:
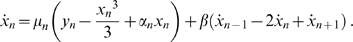
(3)


This additional term represents the expected effect of viscous coupling, in which the dominant force of interaction is the drag that results from the difference between a particular oscillator's velocity and those of the two adjacent oscillators. The parameter *β* determines the strength of this interaction. Equation 3 cannot be simulated directly, but must first be rearranged to
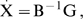
(4a)


in which
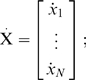
(4b)

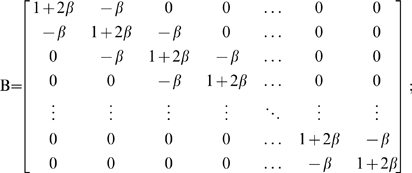
(4c)

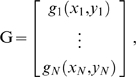
(4d)


with the individual functions *g_n_* representing the right side of Equation 2a.

**Figure 2:**
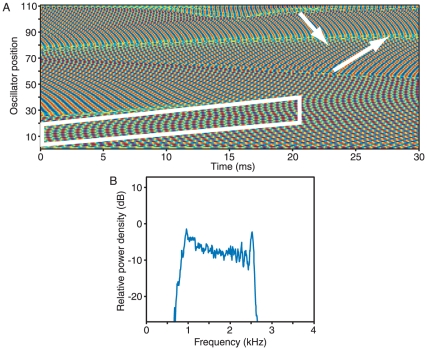
The effect of viscous coupling. A. In this spatiotemporal plot of the system's behavior in the presence of viscous coupling, the abscissa represents time. The ordinate displays the behavior of the 110 coupled oscillators, with those of progressively higher natural frequencies situated toward the top. Colors encode the instantaneous values of the hair-bundle displacement *x*, with red representing positive values, blue negative values, and green values near zero. The oscillators display waves of synchronization moving along the chain in both directions (arrows) and regions of antiphase synchronization (polygon) B. The power spectrum for the same system shows a concentration of power in the frequency range 0.7–2.6 kHz but no well-defined peaks. In this and subsequent figures, the spectral power density is defined relative to that produced by a single, free-running oscillator at its natural frequency. The simulation covered the equivalent of 1.6 s, and the spectrum presented is the average of the power spectra from 47 overlapping segments lasting 128 ms each. The parameter values for the simulations are provided in Table 1.

For positive values of *β*, every element of the matrix **B** ^−1^ has a non-zero value. As a result, the behavior of each oscillator depends in principle on the activity, not just of its immediate neighbors, but instead of the entire ensemble. The system thus experiences an effective global coupling mediated by local interactions, a property that leads to several unexpected behaviors. The most striking phenomenon is the presence of waves of synchronization that advance in both directions along the array (Figure 2A). Note that these waves represent the phase behavior of coupled oscillators: they in no way imply the presence of traveling waves on the basilar membrane! There are also extended periods of alternating antiphase synchronization among oscillators and cyclic motions of defects between phases. These unusual characteristics are reflected in the power spectrum of the simulated emissions (Figure 2B), which differs from experimentally recorded spectra in its lack of well-defined peaks.

### The effect of elastic coupling

Elastic forces could also couple adjacent oscillators. In the gecko, the hair bundles of both tectorial and salletal hair cells are linked by structures with the potential to mediate elastic coupling. Tectorial hair cells insert their hair bundles into a continuous tectorial membrane that encompasses the entire high-frequency region of the basilar papilla; adjacent sallets are conjoined by the longitudinal strand along their top edges. In view of these connections, we next investigated the effect of elastic interactions between hair cells.

We modeled the elastic coupling between successive oscillators by augmenting Equation 2a with a term proportional to the laplacian of position, giving
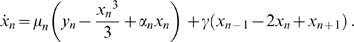
(5)


Here the elastic coupling coefficient *γ* represents the strength of interaction between adjacent oscillators. Except for those at the extremes, each oscillator thus experiences a force proportional to the difference between its position and those of the adjacent oscillators, of which one has a slightly lower and the other a marginally higher natural frequency. Owing to the chain's linear configuration, the outermost oscillators are necessarily subject to asymmetrical forces. Each is linked on one side to the single adjacent oscillator and is connected on the other side to a point whose displacement is fixed at zero (*x*_0_ = *x*_N+1_ = 0). This arrangement inevitably results in smaller oscillations near the ends of the array.

**Figure 3:**
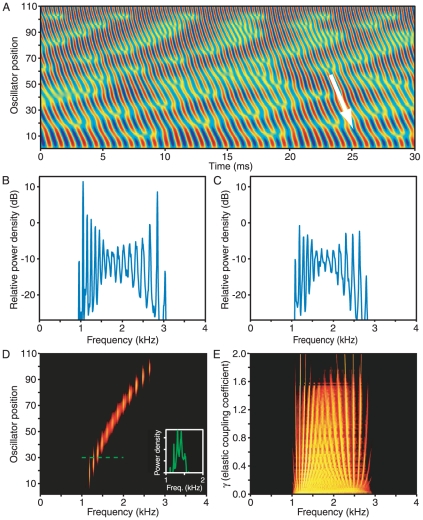
The effect of elastic coupling. A. A spatiotemporal plot portrays waves of synchronization progressing systematically from the high-frequency end of the chain toward the low-frequency end (arrow). Although each oscillator spends most of its time as a component of a group oscillating at a common frequency, the waves are interrupted sporadically by defects as individual oscillators shift from group to group. B. In the power spectrum for elastically coupled oscillators, power is concentrated in 15 central peaks. Each simulation presented in this and the remaining figures covered 3.2 s, divided into overlapping segments of 128 ms. C. A chain in which the amplitude α tapers linearly over the last 18 oscillators at each end yields a power spectrum in which the peaks near the margins are sharper than those near the center. The variation is less pronounced than in (B), however, and more closely resembles that in experimentally recorded spectra. D. Vertically layered power spectra for the 110 individual oscillators indicate that each contributes power to multiple peaks as it shifts its affiliation over time. Colors encode the power-spectral density at each frequency for each oscillator, with brighter areas indicating an increase in density. The inset portrays the power spectrum for the single oscillator indicated by the dashed line. E. Increasing the elastic coupling strength from the bottom power spectrum toward the top causes a progressive reduction in the number of oscillating groups and a sharpening of the group boundaries.

A chain of 110 elastically coupled oscillators readily forms a series of synchronized groups (Figure 3A). The temporal evolution of these oscillators differs qualitatively from that observed with viscous coupling. Waves of synchronized oscillation travel from the high-frequency extreme of the chain toward the low-frequency end, and defects in these waves form when the slope of phases in a small group of oscillators grows unsustainably great. Although these defects appear irregularly during the brief, 30-ms interval represented in the figure, over longer times they can be seen to occur preferentially at certain positions along the chain. As a result, the power spectrum shows distinct peaks that correspond to groups of synchronized oscillators (Figure 3B). Defects occur sporadically within these groups, but they arise more characteristically between the peaks.

The results for this model differ from experimental power spectra in that the peaks near the margins display substantially greater power and less frequency dispersion than those in the chain's middle. In contrast, a model in which *α*, the amplitude of natural oscillations, is constant and equal to 1 along most of the chain, but decreases linearly to 0 over the first and the last 18 oscillators, produces results more similar to those obtained experimentally. The power spectrum for simulation of this system displays localized peaks, but is not dominated by those near the extremes (Figure 3C). We therefore used this pattern of oscillation amplitudes in most of the following simulations.

The power spectra for individual oscillators indicate that most of the power that each produces occurs within a synchronized group, rather than at that oscillator's natural frequency (Figure 3D). This result implies that most oscillators participate in synchronized groups at any given instant. The broad hump of emission in power spectra therefore reflects the superposition of the wide bases of multiple emission peaks, rather than continuum emission from numerous unsynchronized oscillators. The spectra also indicate that each oscillator exhibits power at frequencies corresponding to as many as four different groups, emphasizing the fluid nature of these interactions. The number of synchronized groups is determined primarily by the value of the coupling coefficient *γ*, which fixes both the number of oscillators within each group and the frequency spacing between groups. As *γ* increases, the number of groups declines and they diverge in frequency (Figure 3E). This process occurs through a series of abrupt transitions in which the frequencies of groups near the center of the chain are reorganized, resulting in one fewer groups. At any instant, the oscillation frequency of a synchronized group lies near the median natural frequency of the oscillators within that group. The distribution of frequencies for oscillating groups consequently resembles that of the individual oscillators.

**Figure 4:**
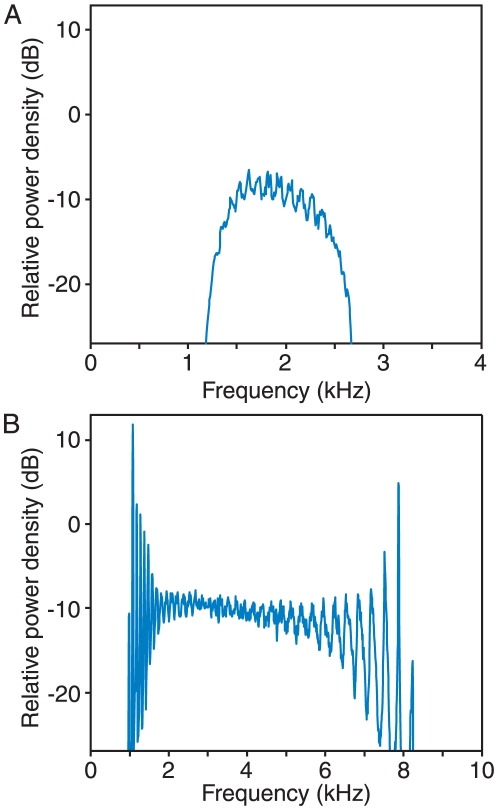
The effect of boundary conditions. A. For a system in which the amplitude of free-running oscillation α varies parabolically across the array, the power spectrum displays poorly defined peaks owing to the absence of sharp boundaries between oscillating groups. B. For a lengthened chain of 210 oscillators, the power spectrum shows sharp peaks produced by oscillators near the boundaries of the basilar papilla, but a poorly defined structure at the frequencies represented by oscillators in the chain's middle. The frequencies are exponentially distributed over an extended range, from 1 to 8.06, owing to the increased length of the chain.

The frequencies of synchronized groups are specified by the presence of symmetry-breaking boundaries in the model. In the simplest case, these boundaries are provided by the ends of the chain of oscillators: the synchronized groups localize in frequency owing to the asymmetrical interactions of the first and last oscillators. As described above, abrupt boundaries lead to dramatic differences between the behavior of groups near those boundaries and those at the array's center, whereas tapered boundaries reduce these distinctions. At the opposite extreme, spectral peaks in the absence of any boundaries become dispersed. Although an infinite chain of oscillators would be necessary to simulate these conditions, the result is mimicked by smoothly varying *α* along a parabola scaled to unity in the center but to zero at both extremes. Such a system lacks sharp boundaries, and as a result the frequencies of synchronized groups are poorly localized and drift continuously over a wide range. The power spectrum therefore shows reduced distinctions between the synchronized groups (Figure 4A). A similar effect can be seen with longer chains of oscillators, for which groups near the middle are distant from the symmetry-breaking boundaries, and again are poorly localized (Figure 4B).

### The effect of structural disorder

**Figure 5:**
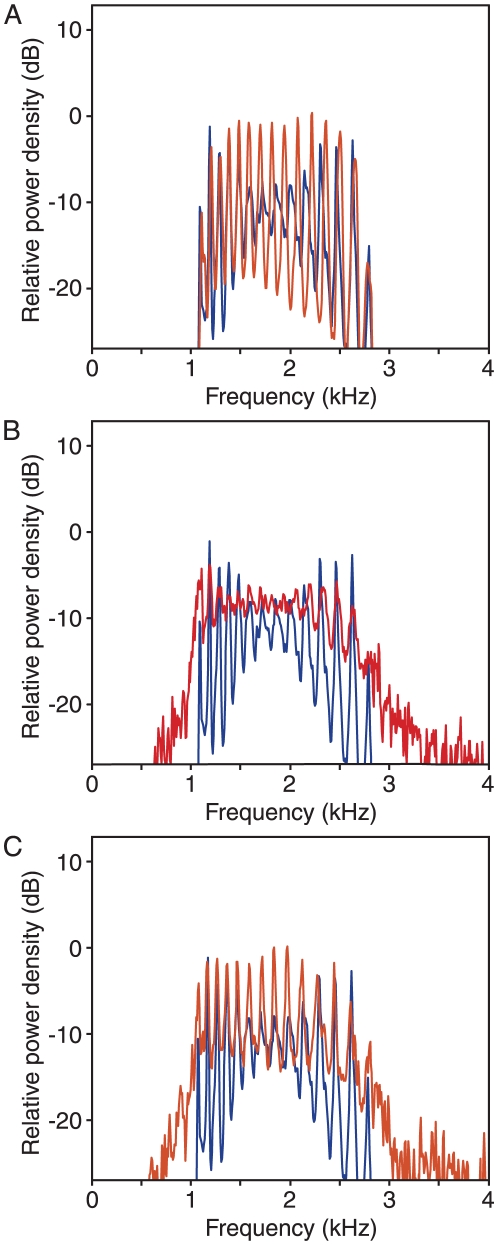
The effects of disorder and noise. A. When all the oscillators are identical, the central peaks of the power spectrum are poorly defined (blue trace). Introduction of random disorder in the value of the free-running amplitude α locks the oscillators into particular groups and thereby sharpens the power-spectral peaks (red trace). In this instance, the value of α over the central 74 oscillators follows a normal distribution with a mean of unity and a standard deviation of 0.03. B. When a control simulation (blue trace) is altered by the addition of white noise, the initially well-defined spectral peaks are effaced (red trace). The noise is approximated by forcing that is normally distributed around 0 with a standard deviation of 0.1 and varies every 16 µs. C. Simultaneous incorporation of noise and disorder shows the reappearance of distinct peaks (red trace).

Our results to this point suggest that the emission spectrum is determined by the boundary conditions for the chain of oscillators. This analysis is clearly insufficient to explain the erratic spacing of emission peaks [45] and the variation between otoacoustic emission spectra recorded from different animals of the same species [17], [18]. Breaking the inherent symmetry of our simple model necessitates variation in some property of the individual oscillators or in the elastic bonds linking them. Introducing slight disorder into the oscillator chain, for example, stabilizes the spectral peaks. An array in which the value of *α* is not constant across the central group of oscillators, but instead varies randomly by a small percentage, displays peaks that are well localized in frequency (Figure 5A). This phenomenon occurs even when *α* deviates by as little as 3% from its average value, a level of disorder far smaller than is likely to exist in the biological system under study. Greater levels of disorder localize the peaks still more precisely and result in spectra with uneven spacing between peaks (data not shown).

### The effect of noise

We simulated the effect of white noise on spontaneous otoacoustic emissions by adding to the right side of Equation 5 a normally distributed random term that varied every 16 µs. In addition to introducing a noise floor in power spectra, this alteration weakens and disperses the most prominent synchronized peaks along the chain (Figure 5B). This effect is opposed to that introduced by structural disorder: when noise and disorder are simultaneously present, well-defined peaks reappear (Figure 5C). We do not know how the amount of noise required to destabilize peaks in our simulations compares to that present in a gecko's natural environment.

### The effect of acoustic stimulation

The spectrum of spontaneous otoacoustic emissions changes in characteristic ways when an ear receives an external stimulus tone. Most notably, the spectral peaks with frequencies near that of the tone usually decline in amplitude and shift in frequency away from that of the stimulus [38], [44]. To test the ability of our model to replicate these effects, we introduced into the model a sinusoidal force acting on all the oscillators. We altered Equation 5 to
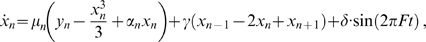
(6)


in which *δ* represents the velocity and *F* the frequency of an acoustic stimulus.

**Figure 6:**
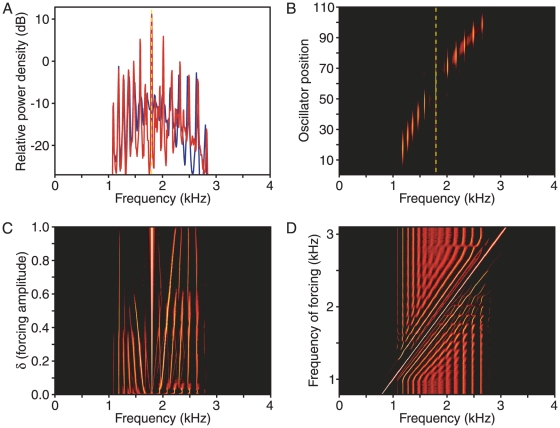
The effect of external stimulation. A. A simulated power spectrum in the absence of stimulation displays relatively weak peaks in its central region (blue trace). The introduction of sinusoidal forcing at a frequency of 1.8 kHz (dashed yellow line) accentuates the central peaks and shifts their frequencies away from that of the stimulus (red trace). The baseline power density immediately surrounding the stimulus frequency is also suppressed; in this instance, the power falls by an average of 12 dB within the 30-Hz-wide band immediately adjacent to the stimulus. B. In the presence of stimulation at 1.8 kHz (dashed yellow line), the power spectra for individual oscillators display sharply defined groups near the frequency of forcing. C. As the forcing amplitude *δ* increases from the bottom of the figure toward the top, vertically layered power spectra reveal the systematic migration of the oscillation frequencies of other groups away from that of the stimulus of 1.8 kHz. D. As the external stimulus frequency is varied over a wide range, as denoted by the line running diagonally from the lower left to the upper right, vertically layered power spectra demonstrate the reorganization of successive groups of oscillators. As the stimulus frequency shifts toward higher values, oscillating groups of higher frequency abruptly disappear one by one, whereas additional lower-frequency groups appear.

In the presence of stimulation, oscillators with natural frequencies near that of the external input become synchronized with it (Figure 6A). As a result, the baseline power at frequencies near the stimulus is suppressed. The external force breaks the symmetry of the chain much as do the boundary conditions examined earlier: the spectral peaks at frequencies near that of stimulation become more pronounced than in the absence of stimulation. Because a group of oscillators is synchronized exactly with the external input, stimulation effectively divides the chain of oscillators into two segments with negligible communication. The power spectra for individual oscillators show a strong, narrow peak at the stimulus frequency (Figure 6B). Adjacent peaks also become higher and better localized owing to their proximity to this imposed boundary.

As the strength of the external stimulus grows, the frequency range over which oscillators become synchronized increases (Figure 6C). At the same time, the power spectrum of the summed oscillators progresses through several regimes. A weak external force induces a rearrangement of the frequencies of spectral peaks; further increases in stimulation cause a repulsion of peaks with frequencies near that of the external force. This counterintuitive behavior results from changes in the population of oscillators that contribute to the repulsed peaks. Increasing the amplitude of the external force causes oscillators with natural frequencies at ever greater distances to synchronize with the input. As a result, these oscillators lose synchronization with their original groups. The frequency of the median oscillator within each of these deprived groups therefore moves further from that of the external force.

Varying the frequency of external stimulation provides further insight into the system's behavior (Figure 6D). For a fixed magnitude of the external stimulation, the bandwidth of oscillators that become synchronized remains constant as the frequency changes. The spectral peaks adjacent to that of the external forcing therefore stay separated by a fixed frequency interval. As the frequency of the external force increases, synchronized groups whose oscillation occurs at frequencies between that of the forcing and that at the chain's high-frequency end are compressed in frequency. When the spacing between adjacent groups drops below a critical value, the entire segment of the chain undergoes a rearrangement, resulting in one fewer synchronized groups. A complementary pattern emerges at frequencies below that of the external forcing. After each rearrangement, the remaining synchronized groups recur at the same locations, providing further evidence that the size of a stably synchronized group is tightly bounded both above and below.

## Discussion

### Mechanical coupling between hair bundles

The spontaneous otoacoustic emissions of the tokay gecko are distinctive in the large number and relatively even spacing of their spectral peaks. This pattern might be thought to originate from the unusual anatomy of the apical portion of the basilar papilla, in which hair cells along half of the organ are covered by a large number of discrete sallets, rather than by a continuous tectorial sheet. We therefore examined a model in which adjacent oscillators interact through the drag forces that might plausibly couple adjacent sallets. Surprisingly, this approach failed to reproduce the pattern seen in experimental emission spectra. In this configuration, the oscillators do not organize into well-delineated, synchronized coalitions. Two distinct factors appear to disfavor the formation of such groups. First, although the model incorporates explicitly only the interactions between each oscillator and its immediate neighbors, it instantiates an effective global coupling linking each oscillator to every other. Second, viscous coupling allows adjacent oscillators to synchronize either in phase or in antiphase. This can be seen most simply for a pair of oscillators, in which the return of one displaced oscillator toward the origin pushes the other in the opposite direction [46]. This anti-diffusive behavior, along with the tonotopic variations in frequency, facilitates unlimited dephasing within synchronized chains, and thus the eventual dissolution of any such groups as their extreme members form more stable interactions with the adjacent groups. The result is a rather uniform spectrum lacking well-defined peaks.

We also investigated models in which adjacent oscillators along the basilar papilla are interconnected elastically. In the gecko, such a coupling could be mediated along the continuous tectorial membrane overlying the hair cells on the neural side of the basilar papilla or through the thin strand linking successive sallets on the papilla's abneural side. As measured by comparison of power spectra computed from simulations to those obtained from recordings in sedated animals, a model with elastic coupling satisfactorily reproduces the essential features of the gecko's spontaneous otoacoustic emissions. In particular, the use of an appropriate value for the elastic coupling constant yields simulated power spectra with about a dozen emission peaks, a value in accord with experimental observations [17], [18]. This behavior arises from the opposition between elastic interactions and the chain's tonotopy. In contrast to the result for viscous coupling, the spatially local and position-diffusive nature of elastic coupling favors uniformly in-phase synchronization of neighbors. At the same time, however, tonotopy limits synchronization to finite spatial ranges owing to the increasingly divergent frequencies of more distant oscillators. The result is a fragmentation of the chain into self-synchronized segments oscillating at different frequencies.

Coupling in the papilla is unlikely to be purely elastic, but probably includes partially viscous elements. However, simulations using a combined viscoelastic coupling introduced no new behaviors (data not shown). Either the viscous coupling was too weak to alter the effects of elastic coupling, or it led to the more disordered patterns associated with purely viscous coupling. We therefore conclude that viscous coupling along the papilla is weak compared to the elastic coupling. This finding is consistent with our earlier argument that the modeled system is overdamped, which relies on viscous forces dominating the effects of inertia. The results of our simulations suggest that elastic interactions dominate both inertia and drag, and thus that the system is overdamped but nevertheless ruled by elastic forces.

Elastic interactions between hair bundles occur in many species, so they do not explain the unusual features of the spontaneous otoacoustic emissions in geckos. It is possible that the elastic interactions in the gecko are mediated by only the thin strand connecting the sallets, and are therefore weaker than interactions mediated by a continuous tectorial sheet. Indeed, a previous model of the gecko papilla suggests that although the elastic forces mediated by the tectorial sheet are of sufficient strength to overwhelm the independent activities of each hair bundle, the weaker forces arising from the thin strand that connects sallets allow a greater degree of independence [32]. The weakness of these interactions might then result in the unusually large number of spectral peaks in the gecko's emissions. Additionally, lizard species lacking any tectorial structures produce emissions that often have a large number of spectral peaks [47]. Those cases therefore either require that elastic interactions be mediated through some other structure or that an alternative coupling mechanism be present.

Another possibility is that tectorial hair cells are coupled through pressure changes in the liquid-filled space behind the tectorial curtain, a feature described only in geckos. The ability of a model with tapered ends to replicate experimental emission spectra suggests that the oscillatory activity of hair bundles along the basilar papilla decreases near the organ's ends. This pattern accords with the fact that the tectorial curtain is interrupted at both extremes of the basilar papilla. The liquid in the space behind this curtain is more free to shift in response to movements of the papilla near its ends than in its middle. The tectorial membrane near the ends might therefore provide less opposition to the movement of hair bundles, thus limiting the activity of those bundles relative to those farther from the edges.

### Structure of the model and comparison with previous models

Although our model excluded the effects of mass, the observed behavior of its units resembles that of a powered harmonic oscillator in which the constituents possess a significant mass. Indeed, treating salletal units along the basilar papilla as harmonic oscillators provides satisfactory estimates of their natural frequencies [32]. Moreover, the spontaneous otoacoustic emissions in another lizard, the bobtail skink, have been modeled as the product of a chain of coupled oscillators in which physical inertia plays a significant role [40].

Although it incorporates both viscous and elastic coupling, the previous model [40] does not correspond to our formulation. In fact, elastic coupling in a system that includes mass corresponds in a massless system to coupling that depends unrealistically on the integral of position. Viscous coupling in a mass-containing system, on the other hand, is mathematically equivalent to elastic coupling in a massless system. Indeed, the simulated results presented for a viscously coupled system with mass [40] resemble the present results with elastic coupling.

Because formulations based on different assumptions regarding the significance of mass nonetheless produce similar responses suggests that neither the formation of synchronized groups nor the appearance of such distinctive behaviors as frequency repulsion depends on the exact equations used. We conclude that oscillators in the inner ears of different species may either experience substantial inertial forces and incorporate viscous coupling, or be overdamped and rely primarily on elastic coupling, with similar end results. The distinction between these two models depends on the precise mechanical details of the inner ear within each species, so further experimental studies are necessary to elucidate which formulation governs each system.

### The effect of external stimulation

Perhaps the most distinctive feature of the power spectra for spontaneous otoacoustic emissions, from geckos as well as other species, is the pattern of changes they undergo in the presence of sinusoidal stimuli. Externally applied tones most commonly suppress and repel nearby emission peaks [38], [44], an effect opposite that expected naively for the application of a sinusoidal force to a single oscillator. In agreement with modeling of the skink's cochlea [40], our results suggest that the suppression results from synchronization of nearby oscillators to the tone, decreasing the power of emission at their original frequencies. Peaks adjacent to the stimulus tones are also enhanced in certain cases [17]. The model suggests that the stimuli act in these cases as boundaries, inducing greater synchronization and thus larger emission peaks in nearby oscillators. The narrow area of stimulus frequency-amplitude space in which this behavior is observed experimentally was not seen in our model, and may emerge from the interaction between this effect and the others described here.

The model explains the repulsive behavior as a shift in the affiliation of individual oscillators with synchronized groups. An external tone synchronizes the oscillations in a strip of hair cells, but also leaves the outlying cells to oscillate at frequencies further from that of the applied tone. This same behavior can also account under certain conditions for the frequency attraction that is less commonly seen [17]. By increasing the scope of responses to external tones, this rearrangement might increase the amount of information conveyed centrally and thereby improve frequency discrimination.

### Differences between individual animals

Our model suggests that variations in the spontaneous otoacoustic emissions of individual geckos result from slight irregularities in the properties of the oscillatory units. Variations of only a few percent in the amplitudes of free-running oscillators suffice to break the symmetry along the chain of oscillators and to localize the frequencies of synchronized groups. These groups coalesce around oscillators with slightly enhanced amplitudes, whereas the discontinuities between groups nucleate near weaker oscillators. The ensuing pattern persists for thousands of cycles of spontaneous oscillation.

Disorder of the magnitude represented in our simulations is almost certainly present in actual basilar papillae, in which the number and arrangement of hair cells varies from row to row [19], [20]. This irregularity is therefore able to explain much of the observed divergence between the emissions of individual geckos. Whether this variation plays any physiological role remains uncertain. The model suggests that minor irregularities also cause significant variations in the responses of individual oscillators to external tones by adjusting the sizes and boundaries of synchronized groups of oscillators. Although we have examined this variation in behavior in response to pure tones in an otherwise noiseless environment, equally large variations between individuals may occur in response to more complex and realistic auditory stimuli.

## Methods

### Recording of spontaneous otoacoustic emissions

We used published techniques [18] to record spontaneous otoacoustic emissions from the ears of mature tokay geckos (*Gekko gecko*) under approved research protocol 04042 of the Institutional Animal Care and Use Committee at The Rockefeller University. In brief, we placed an animal sedated with 25 mg·kg^−1^ pentobarbital sodium (Nembutal, Ovation Pharmaceuticals, Inc., Deerfield, IL) in a folded heating pad maintained at 25°C and located in a darkened, double-walled acoustic-isolation chamber. Under these conditions, the animal breathed normally but usually remained dormant for several hours. We inserted a microphone snugly into an acoustic adapter with an opening 5 mm in internal diameter, then sealed the adapter carefully around the animal's ear canal with silicone vacuum grease. Polarized to 200 V, the calibrated, low-noise microphone (4179, Brüel & Kjær, Nærum, Denmark) had a nominal sensitivity of 100 mV·Pa^−1^.

Sound-pressure signals were measured with a preamplifier with a gain of 10× (2660, Brüel & Kjær) and an A–weighted amplifier operated at a gain of 1000× (2609, Brüel & Kjær) and digitized at 20-µs intervals in overlapping, 80-ms segments for a total period of 120 s. After tapering each record with a Hann window and subjecting it to fast Fourier transformation, we computed power spectra and averaged them across the ensemble of records. The power spectra displayed in Figure 1 were processed to remove background noise. Each spectrum represents the difference between that recorded from a living gecko and a control spectrum obtained after the animal's death.

### Modeling studies

We simulated the behavior of various models with Matlab (version 7.1; The MathWorks, Inc., Natick, MA), using the function **ode45** for the solution of stiff ordinary differential equations. Each oscillator was initialized by assigning it a random phase in a cycle of unperturbed oscillation at its natural frequency. After discarding the results from an initial period of equilibration, we summed the simulated values of the hair-bundle displacement across the ensemble of oscillators. These data were divided into a variable number of time segments, each of which was tapered with a Hann window and subjected to fast Fourier transformation. We then computed power spectra and averaged them across the data segments.
